# Rectal Visceral Sensitivity in Women with Irritable Bowel Syndrome without Psychiatric Comorbidity Compared with Healthy Volunteers

**DOI:** 10.1155/2009/130684

**Published:** 2009-09-17

**Authors:** Signe Spetalen, Leiv Sandvik, Svein Blomhoff, Morten B. Jacobsen

**Affiliations:** ^1^Department of Pathology, Ullevaal University Hospital, 0407 Oslo, Norway; ^2^Centre for Clinical Research, Ullevaal University Hospital, 0407 Oslo, Norway; ^3^Department of Psychosomatic and Behavioural Medicine, Rikshospitalet University Hospital, 0027 Oslo, Norway; ^4^Oestfold Hospital Trust, 1603 Fredrikstad, Norway, University of Oslo, 0316 Oslo, Norway and Norwegian University of Life Sciences, 1432 Ås, Norway

## Abstract

*Background.* Psychiatric comorbidity and visceral hypersensitivity are common in patients with irritable bowel syndrome (IBS), but little is known about visceral sensitivity in IBS patients without psychiatric disorders. *Aim.* We wanted to examine rectal visceral sensitivity in IBS patients without comorbid psychiatric disorders, IBS patients with phobic anxiety and healthy volunteers. *Methods.* A total of thirty-eight female, non-constipated IBS patients without psychiatric disorders and eleven female IBS patients with phobic anxiety were compared to nine healthy women using a barostat double random staircase method. The non-psychiatric patients were divided into those with diarrhoea predominant symptoms and those with alternating stool habits. *Results.* The IBS patients without psychiatric disorders had normal visceral pressure thresholds. However, in the diarrhoea predominant subgroup, the volume discomfort threshold was reduced while it was unchanged in those with alternating stool habits. The phobic IBS patients had similar thresholds to the healthy volunteers. The rectal tone was increased in the non-psychiatric IBS patients with diarrhoea predominant symptoms and in the IBS patients with phobic anxiety. *Conclusions.* Non-constipated IBS patients without psychiatric disorders had increased visceral sensitivity regarding volume thresholds but normal pressure thresholds. Our study suggests that the lowered volume threshold was due to increased rectal tone.

## 1. Introduction

Irritable bowel syndrome (IBS) is characterised by chronic abdominal pain associated with defecation or a change in bowel habit that is diagnosed according to the Rome criteria [1–3]. The aetiology is still unknown, but increased visceral sensitivity is an important mechanism [4]. The reasons for this visceral hypersensitivity have been debated, but principally it may be due to peripheral or central alterations of function. A recent study suggests that hypersensitivity in IBS appears to be determined more by psychological factors than by physiological factors [5]. As much research indicates that the visceral sensitivity thresholds are influenced by cognitive and psychological factors [6–8], comorbid psychiatric disorders may be suspected to influence visceral sensitivity thresholds as well. The prevalence of psychiatric comorbidity in IBS patients ranges from 50% to 90% in gastroenterology clinics [9]. Most researchers have not addressed the possible role of concurrent psychiatric disorders for their findings in visceral sensitivity studies. However, it has been reported that increased tolerance to rectal distension after psychological treatment is significantly associated with improved depression in patients with severe IBS [10], while another study indicates that psychopathology does not predict visceral hypersensitivity in IBS [11]. In a previous study we found that IBS patients with comorbid phobic anxiety had decreased rectal sensitivity for the feeling of gas in addition to altered brain processing, as assessed by event-related potentials compared to IBS patients without psychiatric comorbidity [12]. However, it is still unknown if IBS patients with comorbid phobic anxiety differ from healthy volunteers. Further, most studies on visceral sensitivity in IBS patients are based on subsets of referred patients. A higher degree of fatigue and psychological symptoms as well as lower quality of life are reported in IBS patients seen in referral centres versus primary care [13]. 

 Although IBS patients seem to be hypersensitive to visceral stimuli as a group, visceral hypersensitivity is not present in all patients with IBS [14–17]. Patient heterogeneity may be one reason for this discrepancy. Thus, the complexity of confounding factors influencing pain perception and reporting necessitates careful patient description when disease mechanisms are studied. In the present study, we included healthy volunteers and IBS patients of female gender from outside secondary/tertiary care who were without psychiatric comorbidity and had rather uniform (nonconstipated) symptomatology in order to study rectal visceral sensitivity. As psychological factors seem to be important for developing visceral hypersensitivity and IBS, we hypothesised that IBS patients without comorbid psychiatric disorders would be normosensitive in their gut. In addition, because phobic anxiety patients are characterised by an enhanced attentional awareness of situation-specific threats, we hypothesised that these IBS patients would have altered rectal visceral sensitivity when compared to healthy volunteers. We also wanted to compare visceral sensitivity in diarrhoea predominant IBS patients and IBS patients with alternating stool habits, as the results of visceral sensitivity testing in IBS patients with different bowel habits are conflicting [18–23].

## 2. Material and Methods

### 2.1. Subjects

In total, 22 women recruited from the files of collaborating general practitioners and 210 female respondents to a newspaper advertisement were screened for participation in the study. The screening process included mailed screening questionnaires with respect to IBS criteria, a mailed Hospital Anxiety and Depression (HAD) scale [24] to screen for psychiatric disorders and a telephone interview. Eighty-nine subjects who seemed eligible then underwent the following procedures: clinical assessment, screening blood tests, rectoscopy, a double-contrast barium enema or colonoscopy if not performed during the previous two years and a psychiatric examination, which included the diagnostic Mini International Neuropsychiatric Interview [25]. Symptoms, according to the Rome criteria I [1], of at least 1 year's duration were required for inclusion in the study. Only females were included. 

To ensure that the disease was in an active phase of at least moderate severity, both the patient and the physician had to rate the disorder to at least 5 on a 0–10 visual analogue scale (VAS) measuring current global IBS severity, a score of 0 representing no IBS symptoms. Subjects were excluded if they had a constipation predominant IBS subtype, any organic disease of importance, an HAD score >18, a previous history of psychotic disorder, and any current psychiatric disorder in accordance with DSM-IV axis I criteria. 

A total of thirty-eight women suffering from IBS fulfilled the criteria and were included in this study. They were aged 19–49 years (mean, 32.1 years). The IBS patients were classified as having diarrhoea predominant IBS or IBS with alternating stool habits based on bowel frequency according to the Rome criteria I [1]. However, two patients failed to be classified. 

In addition, eleven female IBS patients with comorbid phobic anxiety disorder were included in order to extend our findings from a previous study [12]. The inclusion and exclusion criteria were the same for these patients, with the exception of the comorbid phobic anxiety disorder. They were aged 27–42 years (mean, 33.7 years). Seven of these subjects had a specific phobia (height, snakes, or spiders), two subjects had agoraphobia without panic, and two patients had a nongeneralised social phobia. These patients were also classified as having diarrhoea predominant IBS or IBS with alternating stool habits, but two patients failed to be classified. 

Most IBS patients had consulted a general practitioner about their abdominal complaints at some time. However, only eleven IBS patients without psychiatric disorders (29%) and four IBS patients with comorbid phobic anxiety (36%) had done so during the last year. Five IBS patients without psychiatric disorders (13%) and one IBS patient with comorbid phobic anxiety (9%) had at some time been referred to a specialist. 

A total of nine healthy females, aged 19–27 years (mean, 24.2 years), were enrolled as controls. They had no history or symptoms of somatic or psychiatric disease. Some clinical and psychometric data of the participants are given in Table 1. The participants were informed to avoid all drugs, except for oral contraceptives, for one week before the examinations. 

The study was approved by our regional ethics committee (Ethics Committee in Health Region 2 of Norway), and performed according to the Declaration of Helsinki. 

### 2.2. Volume-Displacement Device and Anal Manometry

A computer driven barostat (Synectics Visceral Stimulator; Synectics, Stockholm, Sweden) was used to inflate a rectal balloon. The balloon was an 8 cm long cylindrical plastic bag, infinite compliant when intrabag volumes were below 500 mL, and tightly fixed at both ends to a multilumen catheter. One lumen with an inner diameter of 3.3 mm was used for inflation of the bag with air (38 ml per second). Another lumen with an inner diameter of 0.8 mm was used to measure pressure within the bag. Three lumens were perfused with saline and were connected to external pressure transducers and a Synectics polygraph. The manometric ports were located 4, 4.5, and 5 cm distal to the caudal end of the barostat bag.

Rectal barostat pressure and volume and anal manometry were continuously registered, and the sampling rate for the barostat-manometry assembly was 32 per second. The lubricated balloon was inserted into the rectum via an anoscope so that the saline-perfused manometry system monitored the pressure in the anal high-pressure zone. The tube was secured in its proper position with tape. To rule out any leak the barostat bag was inflated before use and tested in water.

### 2.3. Experimental Protocol

All experiments were carried out following a minimum 6-hour fast and following the application of one 120 ml Klyx enema (Ferring A/S, docusate sodium 1 mg/ml, sorbitol 250 mg/ml, methylparahydroxybenzoat, propylparahydroxybenzoat, hydroclorid acid, sodium hydroxide, and water). The subjects were placed in the left lateral position in a bed. The examiner was always present, and the information given was standardised in a written protocol. 

Every experiment started with unfolding the balloon to a volume of 200 ml or until the participants reported discomfort. The minimal distending pressure (MDP) necessary to record respiratory variations was then determined, and the barostat pressure was kept constant at a pressure of 2 mmHg above MDP for 15 minutes.

Visceral sensitivity was assessed using a double random staircase [26]. This technique consisted of a computer controlled random application of two identical series of distension stimuli. The amount of each pressure increment was 4 mmHg. Each pressure increment lasted 20 seconds, and the pressure within the rectal balloon was then lowered to a baseline pressure (0 mmHg) for 30 seconds. The subjects were asked to report the first feeling of gas and stool and to press a button (after a signal 5 seconds before the end of the step) when discomfort was experienced. At the discomfort level the subjects rated the intensity of this feeling on a 100 mm visual analogue scale (VAS) ranging from no discomfort (0 mm) to maximal imaginable pain (100 mm). The procedure was stopped when the subjects had reported discomfort three times. The reproducibility of the double random staircase is published elsewhere [26]. 

### 2.4. Data Analysis

Sensory thresholds were expressed as target pressures and volumes. The discomfort threshold was defined as the average pressure or volume of the first three steps on which the subject gave a positive response. The intensity of the discomfort sensation is reported as the mean VAS value. Rectal muscle tone is inversely related to rectal baseline volume. The baseline rectal volume was expressed as the mean 1-minute value of the barostat volume after 13 minutes baseline registration. During the double random staircase procedure the mean volumes at two distensions reached by most of the participants (8 mmHg and 24 mmHg) were also registered.

### 2.5. Statistical Evaluation

For a comparison of means, the unpaired Student's *t*-test was used, with a 5% significance level. Associations between rectal discomfort thresholds and HAD scores, VAS scores measuring global IBS severity, age, day of menstruation, and baseline tone were investigated using the Pearson's correlation coefficient. Missing values were not replaced by any imputing techniques.

The software package SPSS Statistics (SPSS International BV, Chicago, IL) was used for the statistical analyses.

## 3. Results

The results of the visceral sensitivity testing are presented in Table 2. One IBS patient with comorbid phobic anxiety did not complete the visceral sensitivity testing owing to abdominal pain and was excluded from these analyses. Further, some of the IBS patients did not report the first feeling of gas or stool. There were no significant differences in rectal pressure thresholds between IBS patients without comorbid psychiatric disorders and healthy volunteers. However, these IBS patients had significantly lower volume at the discomfort threshold, as compared with healthy volunteers, and the volume at the gas and stool thresholds tended to be reduced (*P* = .085 and *P* = .094, resp). When the IBS patients with comorbid phobic anxiety were compared with healthy volunteers, no significant differences in visceral sensitivity were found. However, they had increased pressure and volume at the gas threshold compared to the IBS patients without comorbid psychiatric disorders. 

Comparison of visceral sensitivity between IBS patients with diarrhoea and alternating stool habits was only done in patients without comorbid psychiatric disorders because of the small sample sizes in the phobic anxiety group. No significant differences in threshold values were found between these IBS subgroups (Table 3), but the diarrhoea predominant IBS patients tended to have lower volume at the discomfort threshold (*P* = .062). Compared with healthy volunteers, the volume at the stool and discomfort thresholds was significantly reduced in the diarrhoea predominant patients. There were no significant differences between the IBS patients with alternating stool habits and the healthy volunteers.

For technical reasons, baseline rectal volume was not recorded in four IBS patients without psychiatric disorders and in two IBS patients with phobic anxiety. Baseline rectal volume was reduced in IBS patients without psychiatric comorbidity and patients with comorbid phobic anxiety, as compared with healthy volunteers (Table 4). The diarrhoea predominant subgroup had reduced rectal baseline volume, but the subgroup of IBS patients with alternating stool habits was not significantly different from healthy volunteers. The volume at the distension of 24 mmHg was lower in the diarrhoea predominant IBS patients than that in the healthy volunteers, and this volume tended to be reduced in the whole group of IBS patients without comorbid psychiatric disorders (*P* = .062) and in the IBS patients with comorbid phobic anxiety (*P* = .084). 

Correlations were examined in the IBS patients without comorbid psychiatric disorders. There were no significant correlations between rectal discomfort thresholds and HAD anxiety and depression scores, VAS scores measuring global IBS severity, age or day of menstruation. However, the baseline rectal volume was correlated with the volume at the discomfort threshold (*r* = 0.59, *P* < .001) and with the volumes at the distensions of 8 mmHg (*r* = 0.67, *P* < .001) and 24 mmHg (*r* = 0.75, *P* < .001). There were no significant correlations between baseline rectal volume and the pressure at discomfort threshold.

## 4. Discussion

The main purpose of the present study was to examine rectal visceral sensitivity in a well-described population of nonconstipated female IBS patients from outside secondary/tertiary care centres who were without comorbid psychiatric disorders. Compared with healthy volunteers they were hypersensitive in their rectum as far as volume thresholds were concerned, but the pressure was normal at the discomfort threshold. When these IBS patients were subtyped by predominant bowel pattern, the volume discomfort threshold was reduced in patients with diarrhoea predominant symptoms while it was unchanged in those with alternating stool habits.

Discrepancy between pressure and volume thresholds has been described earlier [20, 27], and it has been demonstrated that after prolonged isobaric distension, the volume increased but the perception did not [28]. It is still under debate what the relevant stimuli for the intestinal mechanoreceptors are. There is some evidence that pressure [27] and tension [29] are better candidates than volume, and volume thresholds seem to be more vulnerable to measurement errors than pressure thresholds [30]. However, an experimental study in animals indicates that volume is an important stimulus [31]. This study showed that specialised intranglionic laminar endings in the rectum are appropriate for triggering rectal sensations in response to distension and that their elongation as well as active contraction of the smooth muscle enhanced firing in the afferent nerves [31]. Further, one study indicates that volume thresholds are more reproducible than pressure thresholds [32]. Therefore, we have used changes in pressure, volume, or both as indicators of visceral sensitivity. 

The IBS patients without comorbid psychiatric disorders had reduced baseline volume and tended to have reduced volume at a chosen fixed pressure (24 mmHg) reached by most of the participants. When the IBS patients were subtyped into diarrhoea predominant IBS and IBS with alternating stool habits, these volumes were only significantly reduced in the diarrhoea predominant patients. Further, the baseline volume and the volume at discomfort threshold were significantly correlated. Our findings may indicate that the reduced volume at discomfort threshold in IBS patients is due to increased rectal tone. Increased rectal tone in IBS patients has been reported earlier [33, 34], but not in all studies [35]. Our results are in line with previous studies showing that drug induced [36] or rate dependent [37] rectal contraction enhances rectal perception. Our study cannot explain if the increased rectal tone is due to central or peripheral alteration.

Visceral hypersensitivity has been claimed to be a biological marker of IBS [14]. Some argue that this phenomenon may be explained by psychological bias [5–8, 34]. On the other hand, it is reported that the increased frequency of sensations reported by IBS patients is not due to a psychological response bias [38]. We did not find any association between psychological test scores and visceral thresholds. Some authors report such an association [14, 34], but the majority do not [7, 15, 39]. Studies comparable to our, involving nonpsychiatric IBS patients only, report increased visceral sensitivity in IBS [40–42]. 

Although visceral sensitivity, measured as volume thresholds, was increased in the IBS patients without psychiatric disorders, the pressure thresholds were normal. Most studies report lowered rectal pressure thresholds in IBS patients [14, 16, 17, 41, 42]. There are several possible explanations for this discrepancy. First, because our study included a relatively small number of participants, it is not possible to exclude a type II error in the pressure measurements. However, the observed pressure difference at the discomfort threshold is small and hardly of clinical importance. Second, the IBS patients in this study were older than the healthy volunteers. Older people appear to be less sensitive than younger [43]. The difference in age may have attenuated the difference in the pressure discomfort threshold. However, it is highly unlikely that a mean difference in age of only eight years is of clinical importance, and we found no significant correlation between age and the discomfort threshold. Third, the double random staircase method is supposed to be less biased from psychological influences than the ascending method of limits which is used by most authors. Therefore, it may be argued that our choice of methodology can explain why the discomfort pressure threshold seemed to be normal in our IBS patients. Fourth, the exclusion of psychiatric comorbidity in our IBS patients may have reduced the psychological bias to the response compared to studies including IBS patients with such comorbidity. However, comorbid phobic anxiety did not influence the rectal discomfort threshold, but little is known about visceral sensitivity in IBS patients with other psychiatric disorders. Finally, it can be suspected that our recruitment of IBS patients from outside secondary/tertiary care may have been important. Although most of our IBS patients are recruited by advertisements, they seem to be comparable with nonpsychiatric IBS patients met in general practice. The small number of patients referred to specialists among our IBS patients is consistent with that. We have only been able to identify one study of rectal sensitivity in community IBS patients [44] although only 17–30% of IBS patients in general practice are referred to specialists [45, 46]. In that study diarrhoea predominant, nonpsychologically disturbed, community patients with IBS had normal rectal sensitivity although they had increased gastric perception [44]. A few authors have recruited IBS patients mainly or partly by advertisements, but the consultation habits of their IBS patients are not reported [5, 18, 40].

We did not find any significant differences in visceral sensitivity between diarrhoea predominant IBS patients and IBS patients with alternating stool habits, but the volume at discomfort threshold tended to be lower in the diarrhoea predominant patients. Compared with healthy volunteers, baseline volume and the volume at stool and discomfort thresholds were significantly reduced in the diarrhoea predominant IBS patients. Lower colorectal volume at the discomfort threshold in diarrhoea predominant IBS patients is consistent with the previous studies [19, 20], but lower pressure at discomfort or pain thresholds has been reported as well [18, 19]. Additionally, some studies do not find any evidence of disturbed visceral sensitivity in these patients [22, 23]. In contrast to our findings, it has been claimed that IBS patients with alternating stool habits are hypersensitive [18, 19].

Our previous study indicated that phobic anxiety may modify the gas threshold in IBS [12]. The present study did not show any differences in visceral sensitivity between the IBS patients with comorbid phobic anxiety and healthy volunteers. The gas threshold is far less examined and thought to be less interesting than discomfort and pain thresholds. In a previous study we demonstrated that this threshold seems to be less reproducible than the discomfort threshold in IBS patients [26]. One reason may be the effect of body position on the pressure-volume relationship as described in one earlier study [47]. Therefore, great care was given to standardise the position in these experiments. 

There are several methodological difficulties that arise when assessing visceral sensitivity. Both the psychological context in which a stimulus is perceived and the stable psychological characteristics of the individual influence the perception of pain [6]. In order to reduce cognitive bias, we used a double random staircase method to determine visceral thresholds. We also tried to carefully characterise the IBS patients psychologically. Each visceral testing was performed between 8 AM and 7 PM. The fact that the tests were not performed at the same daytime may be a weakness since a recent study has documented circadian variation of rectal sensitivity [48]. Further, it is known that rectal sensitivity may change with menstrual cycle [49], but we did not observe any correlation between visceral sensitivity and day of menstruation. The difference in age has been discussed above. Other factors known to be able to influence visceral sensitivity, such as meals, sex, and body mass index, were well controlled in this study.

## 5. Conclusion

Nonconstipated female IBS patients from outside secondary/tertiary care centres and without psychiatric comorbidity had increased rectal sensitivity regarding volume thresholds when tested with a double random staircase method, but normal pressure thresholds. Our study suggests that the lowered volume threshold was due to increased rectal tone. Our findings should be further evaluated in larger studies, but the knowledge that visceral sensitivity may be changed also in nonpsychiatric disturbed IBS patients is important with regard to understanding disease mechanisms in IBS. 

## Figures and Tables

**Table 1:** Clinical and psychometric characteristics of 38 IBS patients without comorbid psychiatric disorders, 11 IBS patients with comorbid phobic anxiety and, 9 healthy volunteers. Values are mean ± SD, if not otherwise stated.

	IBS patients		Healthy volunteers
	Without psychiatric disorders	With phobic anxiety	
Age	32.1 ± 8.1*	33.7 ± 6.1*	24.2 ± 2.8
Body mass index (kg/m^2^)	23.8 ± 4.8	24.6 ± 5.6	22.8 ± 2.3
HAD anxiety	5.8 ± 3.1	7.1 ± 3.4	
HAD depression	3.0 ± 3.4	2.7 ± 1.8	
Duration of present	11.9 ± 10.8	10.1 ± 11.2	
episode (years)			
VAS score	6.0 ± 1.1	6.2 ± 1.2	
(physicians assessment)			
VAS score	6.5 ± 1.5	6.9 ± 1.0	
(self-assessment)			
Diarrhoea-predominant	47%	44%	
Alternating stool habits	53%	56%	

*P < .001 from healthy volunteers.

**Table 2:** Visceral sensitivity of rectal barostat measurements in 38 IBS patients without comorbid psychiatric disorders, 10 IBS patients with comorbid phobic anxiety and, 9 healthy volunteers. Values are mean ± SD; pressure is reported in mmHg and volume in Ml; of the IBS patients without psychiatric disorders only 33 reported the first feeling of gas, 34 the first feeling of stool, and 35 reported the VAS at discomfort threshold. Of the IBS patients with comorbid phobic anxiety only 8 reported the first feeling of gas and 9 the first feeling of stool.

	IBS patients		Healthy volunteers
	Without psychiatric disorders	With phobic anxiety	
*Gas thresholds*			
Pressure	10.3 ± 5.9	17.0 ± 9.0**	12.0 ± 5.7
Volume	63 ± 47	108 ± 82**	98 ± 67
*Stool thresholds*			
Pressure	15.3 ± 6.9	16.0 ± 10.2	16.4 ± 5.8
Volume	100 ± 67	102 ± 88	145 ± 77
*Discomfort thresholds*			
Pressure	29.1 ± 9.3	29.7 ± 8.6	31.6 ± 6.1
Volume	197 ± 70*	205 ± 88	249 ± 67
VAS	6.4 ± 1.8	6.1 ± 1.7	6.9 ± 0.9

*P < .05 from healthy volunteers. **P < .05 from IBS patients without comorbid psychiatric disorders.

**Table 3:** Visceral sensitivity of rectal barostat measurements in 17 diarrhoea predominant IBS patients, 19 IBS patients with alternating stool habits, and 9 healthy volunteers. Values are mean ± SD; pressure is reported in mmHg and volume in Ml; of the diarrhoea predominant IBS patients only 12 reported the first feeling of gas, 16 the first feeling of stool and 14 reported VAS at the discomfort threshold. Of the IBS patients with alternating stool habits only 17 reported the first feeling of stool.

	IBS patients		Healthy volunteers
	Diarrhoea predominant	Alternating stool habits	
*Gas thresholds*			
Pressure	8.7 ± 4.5	11.8 ± 6.3	12.0 ± 5.7
Volume	54 ± 40	69 ± 49	98 ± 67
*Stool thresholds*			
Pressure	14.8 ± 6.6	15.8 ± 7.5	16.4 ± 5.8
Volume	85 ± 60*	108 ± 69	145 ± 77
*Discomfort thresholds*			
Pressure	26.6 ± 9.6	29.7 ± 8.3	31.6 ± 6.1
Volume	168 ± 60**	208 ± 63	249 ± 67
VAS	6.3 ± 1.8	6.4 ± 1.9	6.9 ± 0.9

*P < .05 from healthy volunteers. **P < .01 from healthy volunteers.

**Table 4:** Rectal baseline volume (tone) and volume at the distension of 8 mmHg and 24 mmHg in 38 IBS patients without comorbid psychiatric disorders (subdivided into IBS patients with diarrhoea and alternating stool habits), in 11 IBS patients with phobic anxiety and in 9 healthy volunteers. Values are mean ± SD; pressure is reported in mmHg, volume in Ml; the baseline volume was only registered in 34 IBS patients without psychiatric comorbidity and 9 IBS patients with comorbid phobic anxiety. The volume at 8 mmHg distension was only registered in 10 IBS patients without comorbid phobic anxiety. The volume at 24 mmHg distension was only registered in 34 IBS patients without psychiatric comorbidity, 10 IBS patients with comorbid phobic anxiety, and 8 healthy volunteers.

	IBS patients without psychiatric disorders			IBS patients with phobic anxiety	Healthy volunteers
	All	Diarrhoea predominant	Alternating stool habits		
*Baseline*					
Pressure	7.5 ± 1.3	7.8 ± 1.7	7.5 ± 0.9	7.4 ± 1.3	7.2 ± 1.2
Volume	79 ± 48*	60 ± 34**	91 ± 56	50 ± 28**	127 ± 59
*8 mmHg distension*					
Volume	46 ± 40	43 ± 38	48 ± 44	43 ± 32	66 ± 37
*24 mmHg distension*					
Volume	171 ± 59	150 ± 43**	179 ± 60	171 ± 44	216 ± 58

*P < .05 from healthy volunteers. **P < .01 from healthy volunteers.
